# George Carpenter

**Published:** 1910-04-02

**Authors:** 


On Easter Day, suddenly, at the residence of his mother, Coldharbour, Waddon, Croydon, George Carpenter, Esq., M.D.Lond., of 12 Welbeck Street, Cavendish Square, London, W. Dr. Carpenter obtained his medical education at St. Thomas's and Guy's Hospitals, qualifying in 1885 by becoming a Licentiate of the Society of Apothecaries. Three years later he became a member of the Royal College of Physicians, and in 1890 he graduated M.D. of London University. He was physician to the Evelina Hospital, a well-known writer on diseases of children, and for many years ably edited *Pediatrics*. He was also physician to the Queen's Hospital, Hackney, and founder and editor of the *British Journal of Children's Diseases*.

